# Direct Correlation of Surface Tension and Surface Composition of Ionic Liquid Mixtures—A Combined Vacuum Pendant Drop and Angle-Resolved X-ray Photoelectron Spectroscopy Study

**DOI:** 10.3390/molecules27238561

**Published:** 2022-12-05

**Authors:** Ulrike Paap, Vera Seidl, Karsten Meyer, Florian Maier, Hans-Peter Steinrück

**Affiliations:** 1 Department of Chemistry and Pharmacy, Physical Chemistry II, Friedrich-Alexander-Universität Erlangen-Nürnberg (FAU), Egerlandstr. 3, 91058 Erlangen, Germany; 2 Department of Chemistry and Pharmacy, Inorganic Chemistry, Friedrich-Alexander-Universität Erlangen-Nürnberg (FAU), Egerlandstr. 1, 91058 Erlangen, Germany

**Keywords:** ionic liquids, angle-resolved X-ray photoelectron spectroscopy (ARXPS), surface composition, pendant drop (PD), surface tension, ultra-high vacuum (UHV)

## Abstract

We investigated the surface tension and surface composition of various mixtures of the two ionic liquids (ILs) 1-methyl-3-octyl-imidazolium hexafluorophosphate [C_8_C_1_Im][PF_6_] and 1,3-*bis*(polyethylene glycol)imidazolium iodide [(mPEG_2_)_2_Im]I in the temperature range from 230 to 370 K under ultraclean vacuum conditions. The surface tension was measured using a newly developed apparatus, and the surface composition was determined by angle-resolved X-ray photoelectron spectroscopy (ARXPS). In the pure ILs, the alkyl chains of [C_8_C_1_Im][PF_6_] and the PEG chains of [(mPEG_2_)_2_Im]I are enriched at the IL/vacuum interface. In the mixtures, a strong selective surface enrichment of the alkyl chains occurs, which is most pronounced at low [C_8_C_1_Im][PF_6_] contents. For the surface tension, strong deviations from an ideal mixing behaviour take place. By applying a simple approach based on the surface composition of the mixtures as deduced from ARXPS, we are able to predict and reproduce the experimentally measured temperature-dependent surface tension values with astonishingly high accuracy.

## 1. Introduction

Ionic liquids (ILs)—salts with melting points typically well below 100 °C—have raised great interest in research and industry over the last two decades [1,2,3,4,5,6,7]. Apart from other benefits, it is the sheer uncountable possibility of adapting their physicochemical properties by adequate and independent choice among many available cations and anions with specific molecular sub-units, which gained ILs the term “designer solvents” [8,9]. An extension of this design concept is to mix two (or even more) pure ILs [10,11,12]. While the preparation of such a new IL system by mixing known ILs is rather simple, the resulting properties of the mixture often strongly deviate from those of the individual ILs and do not follow ideal mixing rules. These deviations are related to the complex interplay of different short- and long-range interactions between the ions in the mixture [10,11]. It is known that these interactions lead to rather complex structures on the nanoscale in the bulk of pure ILs and IL mixtures [13,14]. The situation for ions at interfaces of the IL with a solid or with the gas phase is even more difficult to predict. A typical example is mixtures containing fluorinated chains, where a preferential enrichment of these moieties at the surface has been reported [15,16,17,18].

In the present study, we focus on the surfaces of IL mixtures, particularly on the resulting surface tension and its relation to the surface composition. While the surfaces and interfaces of pure ILs under the well-defined conditions of ultrahigh vacuum have been studied quite extensively by several groups, including our own [6,19,20,21,22,23,24,25,26,27,28,29], the investigation on the surface composition of IL mixtures with surface science techniques is a rather new field [17,25,30,31,32,33,34]. One important quantity related to liquid surfaces is surface tension (ST), which is the macroscopically observable change in free energy per change in unit surface area. On the microscopic level, the ST originates from the fact that molecules in bulk and at the surface, that is, the boundary to vacuum (or a gas phase), have very different local environments and, thus, different interactions. From this point of view, the ST of ILs has been in the focus of many experimental and theoretical groups [35,36,37,38]. Moreover, the ST also is most relevant for many applications of ILs, including wetting and coating, gas adsorption and release, and multiphase catalysis phenomena occurring at the liquid-gas interface, to name a few (see also the extensive review provided by Tariq et al. and references therein [37]).

The break in symmetry at the surface of a liquid can lead to very distinct preferential orientation and segregation effects of molecules close to the liquid-gas interface, as already discussed at the beginning of the last century by Hardy [39] and Langmuir [40,41]. When studying a series of fatty acid solutions in water, Langmuir observed that at lower concentrations, the hydrocarbon chains lie flat on the water surface. As the concentration of the solution increases, the fatty acid molecules in the surface layer become more densely packed until the surface is saturated by the hydrocarbon chains arranged vertically while the ST levels off [41]. These early findings demonstrate that changes in ST often are very different from changes in bulk composition, that is, the ST cannot be derived from ideal mixing rules. In his review “Forces Near the Surfaces of Molecules”, Langmuir also reported that for all pure organic liquids comprised of molecules with long alkyl tails (such as *n*-hexane and *n*-hexanol), the surface tensions are virtually equal and, thus, independent of the hydrocarbon length. He deduced that the forces along the entire length of the alkyl part of the molecule are almost uniform. To explain the ST values of aliphatic alcohols, Langmuir split the ST into independently acting contributions of head (that is, the hydroxyl group) and tail group (hydrocarbon chain) [42]: for the long-tail liquids, the tails are predominantly covering the outer surface (and thus, very similar ST values are measured) while for liquids with shorter alkyl chains, the hydroxyl groups are also significantly exposed to the gas phase leading to changes in ST. In this simple picture, the ST of these hydrocarbons is thus ascribed as the result of independent contributions from the molecular units (Langmuir called them “parts of molecular surfaces”) that are exposed on average to the outer surface [42,43].

Following these ideas, we already employed this “Langmuir principle” successfully for a series of pure *n*-alkyl-imidazolium ILs with increasing chain length n. By combining structural data obtained from angle-resolved X-ray photoelectron spectroscopy (ARXPS) experiments with molecular dynamics (MD) simulations, we were able to predict the correct surface tension trend along this IL series [44]. We also performed a first study addressing mixtures of ILs with the common [PF_6_]^−^ anion and imidazolium cations carrying either short butyl or partially fluorinated butyl chains [15,45] by combining conventional ST measurements (pendant drop measurements under 1 bar argon atmosphere) and ARXPS. Very recently, a similar approach was successfully employed by the McKendrick group using reactive-atom scattering with laser-induced fluorescence (RAS-LIF) in combination with MD and ST measurements [17,18]. From their RAS-LIF signals that originate mainly from alkyl units located at the outer surface, the authors derived a quantitative analysis of the alkyl content at the surface, which allowed them to describe the observed ST values of the mixture using the Langmuir concept as described above. They studied mixtures of ILs with different alkyl chain lengths in the cation and also with partially fluorinated chains at room temperature. From the underrepresentation of the alkyl signals, the authors could indirectly deduce a preferential surface enrichment of the fluorine chains in the mixtures [17,18].

**Figure 1:**
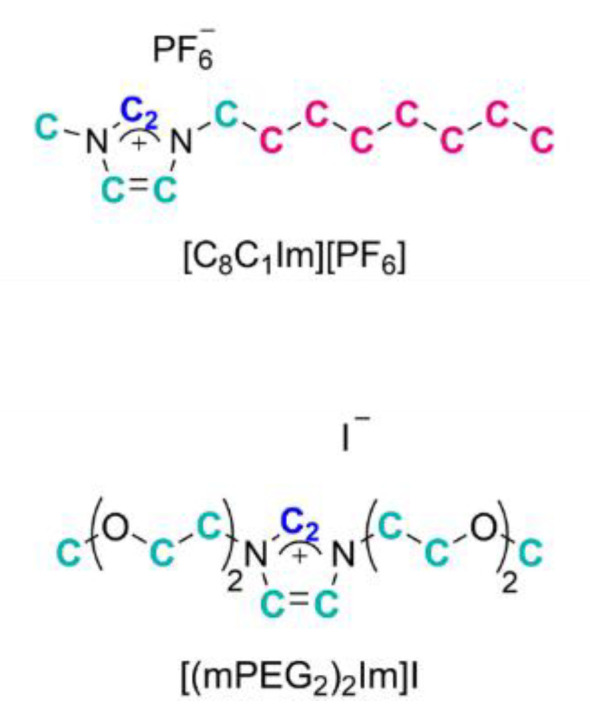
Structure of 1-methyl-3-octyl-imidazolium hexafluorophosphate [C_8_C_1_Im][PF_6_] (**top**) and 1,3-bis(polyethylene glycol)imidazolium iodide [(mPEG_2_)_2_Im]I (**bottom**). The different carbon atoms giving rise to the three discernible C 1s peaks are color-coded in blue (C_2_ peak), in turquoise (C_hetero_), and in pink (C_alkyl_).

In the present work, we now extend this concept to IL mixtures with very different anions and cations. The corresponding measurements of the temperature-dependent ST were performed under ultraclean vacuum conditions with an apparatus recently developed in our lab [46], thereby minimizing any risk of possible surface contamination affecting the ST. The surface composition was determined from temperature-dependent ARXPS in an ultra-high vacuum [26,47,48,49]. ARXPS allows, in particular, for verifying the purity of the ILs and the analysis of all elements (but hydrogen) present in the IL mixtures. In particular, we studied the pure ILs [(mPEG_2_)_2_Im]I and [C_8_C_1_Im][PF_6_], and their mixtures with about 5, 10, 20, and 50 mol% [C_8_C_1_Im][PF_6_] content at temperatures between 230 and 370 K. The two ILs have different anions (I^−^ and [PF_6_]^−^) and imidazolium-based cations with long functionalized and non-functionalized chains (see Figure 1), allowing for very different types of interactions in the mixtures. Both ILs were also selected due to (a) their rather wide liquid temperature window, (b) the existing knowledge on surface composition and surface tension of the pure ILs [31,37,50,51], and (c) the fact that each ion contains atoms that can be unambiguously assigned in the ARXP spectra. For the mixtures, we deduce a clear preferential surface enrichment of the [C_8_C_1_Im]^+^ cations from the octyl chains’ XPS signals and a concomitant depletion of the [(mPEG_2_)_2_Im]^+^ cations from the XPS signals of the polyethylene glycol (PEG) chains. This non-stoichiometric surface behaviour is reflected in the measured surface tension values of the mixtures. We demonstrate that the simple Langmuir concept allows for linking surface tension and surface composition of IL mixtures in a quantitative way, even for very complex ions. Based on the surface composition and the temperature-dependent ST data of the pure ILs, we can predict the ST of IL mixtures from their surface composition as determined by ARXPS. As a reverse conclusion, the ST value measured for an IL mixture at a certain temperature under clean conditions (ruling out surface impurities) allows for deriving the composition at the surface.

## 2. Results and Discussion

### 2.1. ARXPS

**Figure 2:**
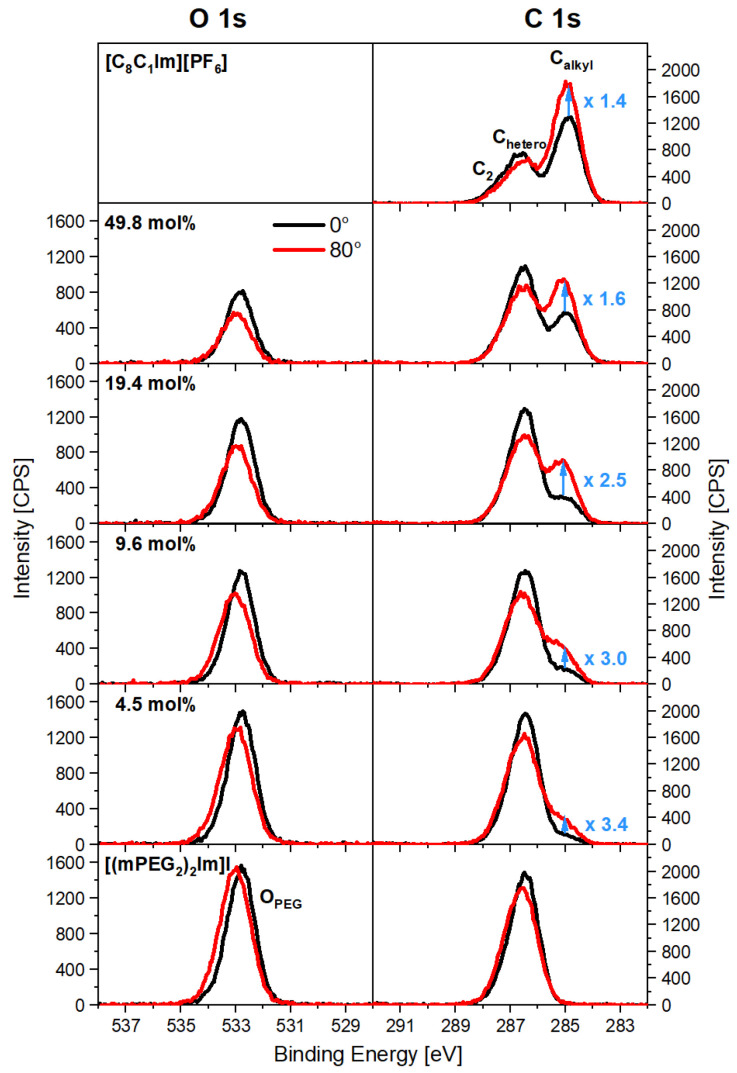
O 1s and C 1s spectra at 0° (black) and 80° (red) emission of pure [C_8_C_1_Im][PF_6_], pure [(mPEG_2_)_2_Im]I, and of different mixtures of these ILs, collected at T = 298 K. The relative increase in C_alkyl_ intensity upon changing the emission angle from 0° to 80° is indicated (for details, see text).

We performed temperature-dependent ARXPS measurements for the pure ILs 1-methyl-3-octyl-imidazolium hexafluorophosphate [C_8_C_1_Im][PF_6_] and 1,3-*bis-*(polyethylene glycol)imidazolium iodide [(mPEG_2_)_2_Im]I (see Figure 1) and for different mixtures of the two ILs with 4.5, 9.6, 19.4, and 49.8 mol% [C_8_C_1_Im][PF_6_] content under ultra-clean UHV conditions at a pressure in the range from 8 × 10^−11^ to 1.5 × 10^−9^ mbar (further details are provided in Section 4 Experimental). Figure 2 shows the O 1s and C 1s regions of these mixtures at 298 K along with the corresponding data of the pure ILs, for emission angles of 0° and 80° (the fitting deconvolution of the C 1s spectra is demonstrated in Figure S1 of the Supplementary Materials for the 49.8 mol% mixture). The spectra of all other species are provided in Figure S2. 

Comparing the bulk-sensitive normal (0°) and the surface-sensitive grazing (80°) emission spectra for pure [(mPEG_2_)_2_Im]I (Figure 2, bottom), we observe a small shift to higher binding energies for the O_PEG_ peak (0.3 eV) and the C_hetero_ peak (0.2 eV) in the surface-sensitive 80° spectra, which is not present for the N and I signals (see Figure S2; for peak assignments, see also colour coding in Figure 1 and Figure S1). This shift is due to the preferential enrichment of the PEG chains at the surface and a related surface core level shift (SCLS) for these chain atoms. This SCLS stems from the different environment of chains at the outer surface as compared to chains in the bulk, as has been discussed previously for other IL mixtures [51]; the effect is less pronounced for the C_hetero_ signal since atoms from the imidazolium ring also contribute to this peak. SCLSs in the O_PEG_ peak are also observed for the mixtures, indicating the presence of PEG chains at the surface, albeit to a lower extent (see below).

The preferential surface presence of the PEG chains in the case of pure [(mPEG_2_)_2_Im]I is also evident from the ARXPS intensities: the O 1s spectra at 0° and 80° show the same height of the O_PEG_ peak but a slightly larger full-width-half-maximum (FWHM) at 80°, which yields 1.1 times larger O 1s intensity (peak area) in grazing emission as compared to normal emission. In the C 1s region, the C_hetero_ peak has a lower peak height but a larger FWHM at 80° compared to 0°, which results in similar intensities at both angles. The larger O_PEG_ and C_hetero_ peak widths at 80° are due to the fact that at this emission angle, the signals originate from the shifted contribution of oxygen or carbon atoms in the outermost layer plus unshifted contributions from underneath, whereas at 0° the signal is strongly dominated by atoms in the bulk. These observations for pure [(mPEG_2_)_2_Im]I are in line with a moderate preferential orientation of the PEG chains towards the surface, which is described in more detail in Ref. [50]. With increasing [C_8_C_1_Im][PF_6_] content in the bulk, we observe for the mixtures a clear decrease in O_PEG_ intensity at 0° in Figure 2, as expected. In contrast to the situation of pure [(mPEG_2_)_2_Im]I, a very pronounced decrease in O_PEG_ intensity is found when changing the emission angle to 80°. This selective surface depletion of the PEG chains becomes more pronounced with increasing [C_8_C_1_Im][PF_6_] content in the mixtures.

For pure [C_8_C_1_Im][PF_6_] (Figure 2, top), the C 1s spectrum at 80° displays a lower intensity of the C_2_/C_hetero_ peaks but a significantly larger intensity of the C_alkyl_ peak as compared to 0°. These findings indicate an orientation of the surface cations with the alkyl chains pointing towards the vacuum and the C_2_ and C_hetero_ atoms towards the bulk, as already observed for many ILs with the [C_8_C_1_Im]^+^ cation [52]. For the mixtures, these enrichment and depletion effects are also present. Notably, the relative C_alkyl_ enrichment at the surface is stronger at lower [C_8_C_1_Im][PF_6_] content (see numbers given next to the C_alkyl_ peaks in Figure 2). At the same time, the C_2_ and C_hetero_ depletion is less pronounced at lower [C_8_C_1_Im][PF_6_] content, which is attributed to the different degrees of enrichment and depletion of the C_alkyl_ and PEG chains in the different mixtures (note again that the two different cations both contribute to the C_2_ and C_hetero_ signals, while C_alkyl_ solely originates from the [C_8_C_1_Im]^+^ cation, see Figure 1).

**Figure 3:**
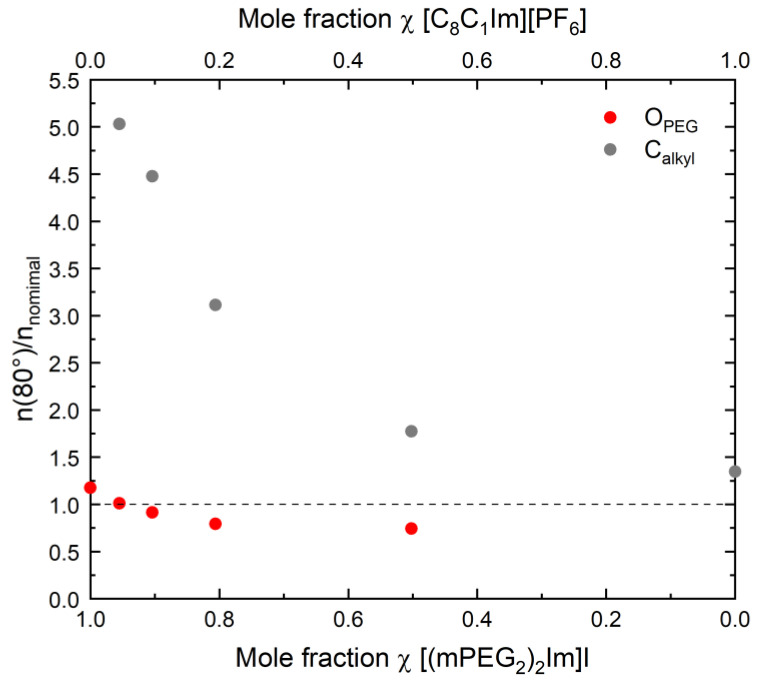
Normalized content n(80°)/n_nominal_ for O_PEG_ (red) and C_alkyl_ (grey), derived from the corresponding 80° intensities and the nominal atomic numbers of O_PEG_ and C_alkyl_ for the pure ILs and mixtures at T = 298 K.

To obtain more detailed insight, we quantitatively analyse the O_PEG_ and C_alkyl_ signals of the pure ILs and the IL mixtures at 298 K. We concentrate on the surface-sensitive measurements at 80°, since they reflect the surface composition of the IL mixtures, which will later be correlated with the macroscopic surface tension. In Figure 3, the normalised O_PEG_ (red circles) and C_alkyl_ (grey circles) contents, that is, the atomic ratios n(80°)/n_nominal_, are plotted, as derived from the corresponding XP spectra at 80°. Notably, a value of 1 (dashed line) would correspond to an isotropic mixture with randomly oriented cations and anions in the bulk as well as at the surface. Figure 3 clearly shows a strong selective presence of the alkyl chains of the [C_8_C_1_Im]^+^ cation at the outer surface at very low [C_8_C_1_Im][PF_6_] contents, yielding an n(80°)/n_nominal_ value of ~5 at 4.5 mol%. With increasing [C_8_C_1_Im][PF_6_] content, the alkyl chain enrichment becomes less pronounced and reaches a value of 1.34 for pure [C_8_C_1_Im][PF_6_] at 298 K. Whereas the moderate enrichment factor of 1.34 is solely due to the orientation of the [C_8_C_1_Im]^+^ cations in the surface layer of [C_8_C_1_Im][PF_6_], the high values for the mixtures are mainly due to preferential surface segregation (plus chain orientation) of the [C_8_C_1_Im]^+^ cations. Simultaneously to the enrichment of the alkyl chains, we observe in ARXPS the depletion of the PEG chains (O_PEG_ signal) with increasing [C_8_C_1_Im][PF_6_] content in the mixtures, from 1.18 for pure [(mPEG_2_)_2_Im]I to 0.71 for the 1:1 mixture with 49.8 mol% [C_8_C_1_Im][PF_6_]. 

**Figure 4:**
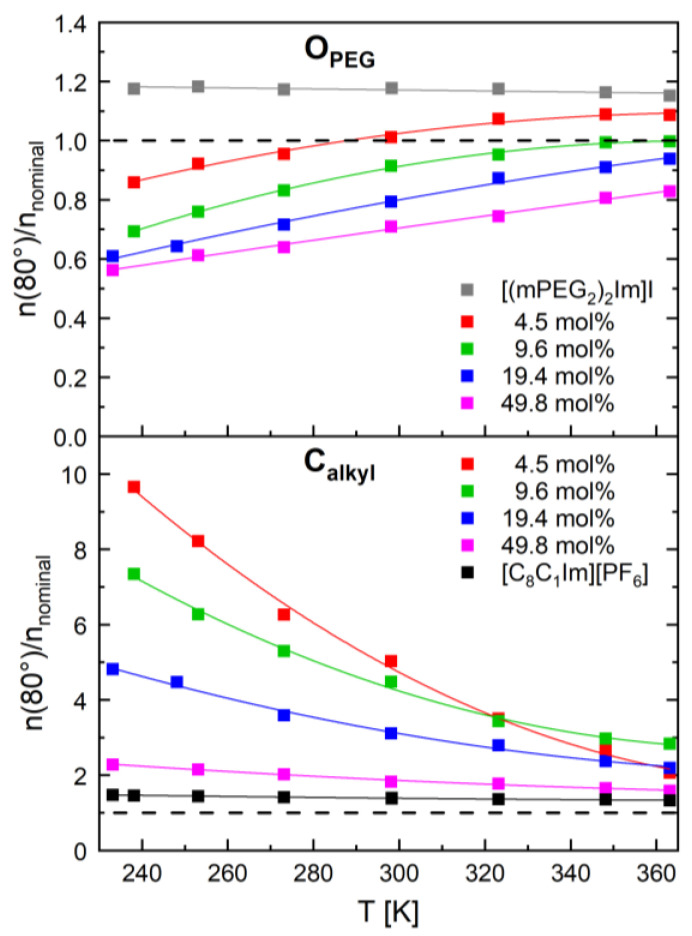
Normalized content n(80°)/n_nominal_ for O_PEG_ (**top**) and C_alkyl_ (**bottom**), derived from the corresponding 80° intensities and their nominal atomic numbers of O_PEG_ and C_alkyl_ for the pure ILs and mixtures for different temperatures (measurements started at 363 K).

Next, we discuss the temperature dependence of surface enrichment and depletion as well as the orientation effects of the mixtures and also pure ILs. The measurements were performed from 363 K down to 238 / 233 K, with the lower temperature depending on the solidification temperature of the samples. The quantitative analysis of the C_alkyl_ and O_PEG_ data is given in Figure 4. For pure [C_8_C_1_Im][PF_6_] (black squares), the n(80°)/n_nominal_ ratio for C_alkyl_ shows a weak decrease from 1.48 at 223 K to 1.32 at 363 K, which is attributed to the fact that the surface [C_8_C_1_Im]^+^ cations are more randomly oriented at higher temperatures. For the mixtures, however, the n(80°)/n_nominal_ ratio for C_alkyl_ shows a very pronounced decrease with increasing temperature. This decrease is attributed to the loss of selective surface enrichment of the alkyl chains relative to the PEG chains (see below), that is, a loss in preferential surface segregation of the [C_8_C_1_Im]^+^ cations at elevated temperatures. It is most pronounced for small [C_8_C_1_Im][PF_6_] contents: e.g., for the 4.5 mol% mixture, the n(80°)/n_nominal_ ratio decreases from around 10 at 238 K down to 2.1 at 363 K.

With increasing temperature, in the case of pure [(mPEG_2_)_2_Im]I the normalised O_PEG_ content (grey squares) at the surface measured at 80° shows virtually no change (see a very weak decrease from 1.17 to 1.15 in Figure 4). In contrast, the mixtures display a clear and continuous increase of the O surface content with temperature, e.g., from 0.86 at 238 K to 1.09 at 363 K for the mixture with 4.5 mol% [C_8_C_1_Im][PF_6_]. This behaviour reflects the increase of the PEG chain contribution at the surface, which goes along with the above-described loss in the alkyl chain signal. As already discussed previously for pure ILs and IL mixtures [15,30], we attribute the generally observed loss in alkyl chain enrichment in ARXPS with increasing temperature to the loss in surface order as an entropic effect.

Finally, all other core level intensities of the imidazolium head groups and the anions show little changes with temperature (for the corresponding n(80°)/n_nominal_ values, see Figure S3 of Supplementary Materials). This is to be expected, as they are preferentially depleted from the outer surface, which is deduced from their values below one. Only the F content of the [PF_6_]^−^ anion increases with increasing temperature to values above 1 for the 4.5 mol% and 9.6 mol% mixtures, which might indicate that [PF_6_]^−^ has a higher surface affinity as compared to I^−^ as the other anionic species.

### 2.2. Pendant Drop Measurements

**Figure 5:**
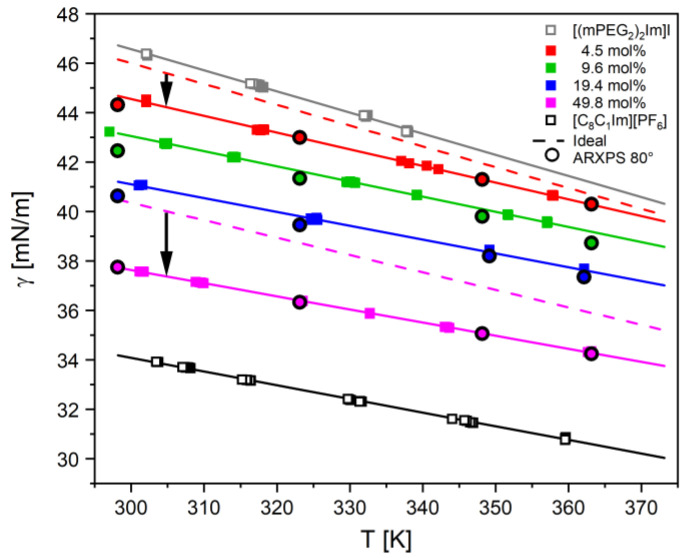
Surface tension values measured using the PD method are shown as open and full squares, along with their linear fits (lines) using Equation (1). The surface tension values calculated from the 80° ARXPS data using Equation (3) are shown as filled circles. Furthermore, the ideal behaviour for the surface tension of the 4.5 and 49.8 mol% mixtures as determined by Equation (2) (dashed lines) is included for comparison (a comparison for all mixtures is shown in Figure S4).

Temperature-dependent pendant drop measurements were performed to determine the surface tension for the above-studied pure ILs and IL mixtures under clean HV conditions at background pressures in the low 10^−6^ mbar range. As in the case of ARXPS, ST data were recorded starting with the highest temperature from 363 down to 300 K, as shown in Figure 5 for the pure ILs (open squares) and the IL mixtures (full squares). For all systems studied, we observe a linear decrease of the ST with increasing temperature, which is fitted by the following equation

(1) $$\gamma_{calc}=\gamma_{0}+\gamma_{1}\cdot T\;$$

where γ_0_ and γ_1_ are the fitting coefficients (see Table 1) and T the temperature in Kelvin. This equation is then used to calculate the ST at the standard temperature of 298 K (Table 1). The density parameters used for ST evaluation are given in Table S1 in the Supplementary Materials.

**Figure 6:**
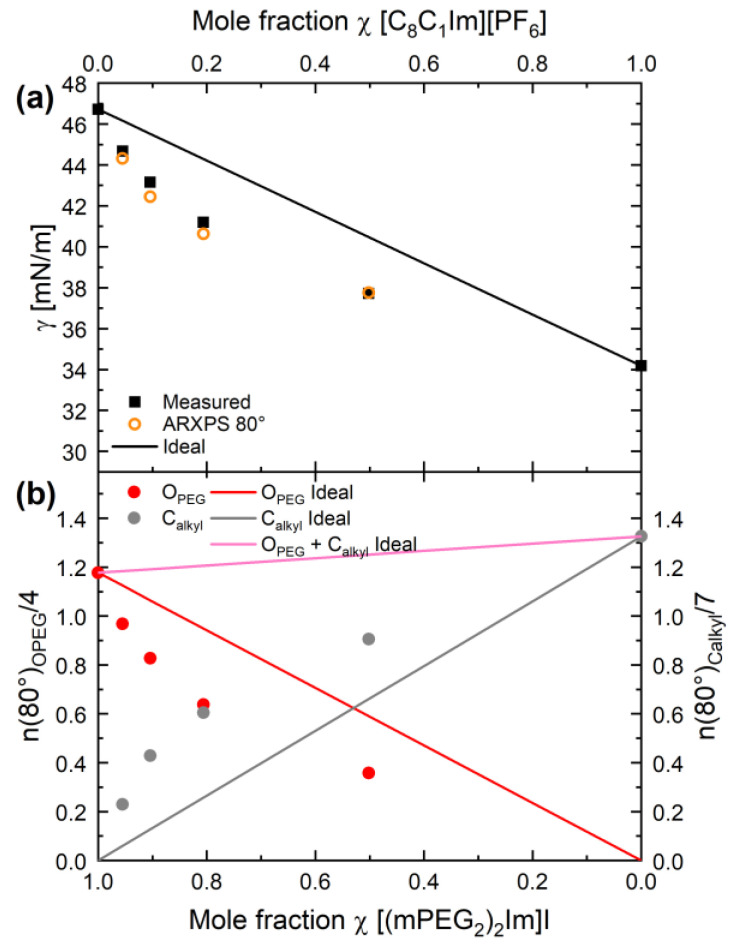
(**a**) Surface tension values of the pure ILs and the mixtures at 298 K (black squares), as determined from pendant drop measurements in Figure 5, along with the surface tension values calculated from the 80° ARXPS data according to Equation (3) (open orange circles) as a function of bulk mole fraction. For comparison, the surface tension of ideal mixtures without enrichment/depletion effects is shown as the black line. (**b**) Number of atoms, n(80°), as determined from the ARXPS data at 80°, normalised to the number of corresponding atoms in the IL for O_PEG_ (red circles) and for C_alkyl_ (grey circles) for the pure ILs and for the mixtures at T = 298 K. Straight lines show ideal behaviour, that is, expected changes without surface enrichment/depletion effects being present.

The ST decreases with increasing [C_8_C_1_Im][PF_6_] content. When comparing the different ST values at 298 K in Table 1 and in Figure 6a (black squares), we find the highest value of γ = 46.72 mN/m for pure [(mPEG_2_)_2_Im]I and the lowest value of 34.18 mN/m for [C_8_C_1_Im][PF_6_], with the values for the mixtures falling in between. Notably, the change in ST is more pronounced at lower [C_8_C_1_Im][PF_6_] contents: it decreases from 46.72 for 0 mol% to 44.67 for 4.5 mol% (by −2.05 mN/m), and further to 43.16 mN/m for 9.6 mol% (by −1.51 mN/m), whereas it only decreases from 37.72 mN/m for 49.8 mol% to 34.18 mN/m for 100 mol% (by −3.56 mN/m); see also Table 1. The same behaviour is observed for all temperatures studied, as is evident from Figure 5. 

The ST of the mixtures thus does not follow an ideal behaviour, which would be obtained from the ST values γ*_i_* of the pure ILs weighted with the corresponding bulk mole fractions *x_i_*:

(2) γT,mixture(x)=γT,[(mPEG2)2Im]I⋅x[(mPEG2)2Im]I+γT,[C8C1Im][PF6]⋅x[C8C1Im][PF6] 

In Figure 5, the non-ideal behaviour is evident from the fact that the fits to the measured data for 4.5 mol% (solid red line) and 49.8 mol% (solid pink line) fall significantly below the ideal characteristics (dashed lines with deviations indicated by black vertical arrows, see also Figure S4 in the Supplementary Materials for all mixtures studied). 

In Figure 6a, an ideal behaviour of the ST would correspond to a linear decrease with increasing [C_8_C_1_Im][PF_6_] content (solid black line). The much stronger decrease than the ideal behaviour at low [C_8_C_1_Im][PF_6_] contents and the fact that the ST values of all mixtures fall below the ideal line is attributed to the strong preferential enrichment and depletion effects occurring at the liquid-vacuum interface. The latter were deduced from the [C_8_C_1_Im]^−^ and [(mPEG_2_)_2_]^−^ chain signals in ARXPS (see above). This deviation between bulk and surface composition then strongly affects the ST, as will be detailed in the next section.

### 2.3. Comparison of ARXPS and PD Results

To obtain further insight, we directly link our ARXPS and PD results. For this purpose, we correlate the ST data for the mixtures at 298 K in Figure 6a to the corresponding atomic O_PEG_ and C_alkyl_ contents in Figure 6b, as determined from the 80° ARXP spectra; the latter are normalised to the number of corresponding atoms in the ion, that is, four O_PEG_ atoms in [(mPEG_2_)_2_Im]I, and seven C_alkyl_ atoms in [C_8_C_1_Im][PF_6_]. In this presentation, the O_PEG_ signal (red circles) represents the surface concentration of the PEG chains of the [(mPEG_2_)_2_Im]^+^ cation, and the C_alkyl_ signal (grey circles) the surface concentration of the alkyl chains of the [C_8_C_1_Im]^+^ cation. When comparing the experimentally obtained data with the linear concentration dependences expected for ideal behaviour (red and grey solid lines for O_PEG_ and C_alkyl_, respectively), we again observe a clear deviation: The measured O_PEG_ data clearly fall below the ideal line and the C_alkyl_ data above the ideal grey line. This reflects the preferential depletion of the PEG chains and the enrichment of the alkyl chains in the mixtures at the surface, as discussed above. 

As the next step, we follow an approach, which has been put forward by us recently for pure ILs along the denoted Langmuir principle [44], which directly relates the ST value of a liquid to the superposition of independent group contributions from the molecular moieties being present right at the liquid/gas interface, that is, at the outer surface. The basic idea now is that we transfer this concept to the binary IL mixtures: the groups of [(mPEG_2_)_2_Im]I and the [C_8_C_1_Im][PF_6_], which are predominantly present at the outer surface—and thus, mainly contribute to the ST of the pure ILs—are used to represent the effective surface mole fraction of the corresponding IL in the mixture. Since the alkyl chains (C_alkyl_ peak) and the PEG chains (O_PEG_ peak) are the dominating surface species in [C_8_C_1_Im][PF_6_] and [(mPEG_2_)_2_Im]I, respectively, we start with only these two signals measured in our most surface sensitive 80° geometry. As it will be shown, this simplification already provides very good agreement with the measured temperature-dependent ST values without further refinement needed. 

Instead of Equation (2) for ideal mixing behaviour, the ST of a mixture with a certain bulk composition *x* at temperature T is estimated from n_OPEG,80°_(x) and n_Calkyl,80°_(x), that is, the number of O_PEG_ and C_alkyl_ atoms, respectively (see Table S2), as derived from the temperature-dependent 80° ARXP spectra:

(3) γT,ARXPS 80°(x)=γT,[(mPEG2)2Im]I⋅nOPEG,80°(x)4+γT,[C8C1Im][PF6]⋅nCalkyl,80°(x)7nOPEG,80°(x)4+nCalkyl,80°(x)7

with γT,[(mPEG2)2Im]I and γT, [C8C1Im][PF6] being the temperature-dependent ST values of pure [(mPEG_2_)_2_Im]I and [C_8_C_1_Im][PF_6_] (Table 1), respectively. 

The XPS-derived ST values of the mixtures using Equation (3) are plotted for selected temperatures (circles for 298, 323, 348, 363 K) in Figure 5 as a function of [C_8_C_1_Im][PF_6_] mole fraction; see also Table 2. The agreement between the calculated and measured ST of the mixtures is extremely good (around 1% deviation with a maximum deviation of 1.7% for one data point). Note that this small deviation is even more surprising since the overall accuracy in IL surface composition determination by ARXPS is at best 5% due to our experience over many years, and thus, further improvement of our model makes little sense. Moreover, we are aware that using this simplified model with two signals only (neglecting, e.g., all other IL components that are also in part present at the outer surface) and the limited surface sensitivity of the 80° geometry [44] makes the agreement between measured and calculated ST even more surprising. Concerning the second argument, the 80° geometry apparently is sensitive enough to account for the deviations in surface mole fraction from the bulk composition. In contrast, this is not the case for the 0° spectra: As a control experiment, we also calculated the ST from the n_OPEG,0°_(x) and n_Calkyl,0°_(x) values derived from the 0° ARXP spectra using Equation (3) (see Tables S2 and S3). The corresponding ST values for 298 K in Figure S5 in the Supplementary Materials and also the temperature-dependent measurements in Figure S6 in the Supplementary Materials show only very small deviations from the ideal behaviour, which is to be expected since the ARXPS signals at 0° with the—around six times—larger information depth as compared to the 80° are in fact dominated by bulk with the small surface contributions only.

At this point, we briefly want to come back to the very recent study by the McKendrick group, who addressed mixtures of ILs with different alkyl chain lengths and mixtures of non-fluorinated ILs with ILs carrying per-fluorinated alkyl chains using an overall very similar approach [17,18]. They employed reactive-atom scattering with laser-induced fluorescence (RAS-LIF) in combination with MD simulations and ST measurements with the pendant drop method under atmospheric conditions, all performed solely at room temperature. Particularly for the mixtures with fluorinated IL chains, the underrepresentation of the alkyl signals in RAS-LIF provided a quantitative measure for the preferential depletion of the alkyl chains and—indirectly—the enrichment of the fluorine chains at the outer surface [17,18]. This allowed the authors to describe the observed ST values of the mixture using the Langmuir concept reported previously [17,18]. When comparing RAS-LIF employed by the McKendrick group and our AXRPS approach, one has to state that both methods are very well suited to monitor the surface composition but have their strengths and weaknesses. RAS-LIF is most sensitive solely to the outermost hydrogenated carbon atoms at the surface, while ARXPS at 80°, as applied here, integrates over the topmost one nm. On the other hand, ARXPS is able to analyse all elements, and thus in our case, we studied the enrichment of the alkyl chains and the depletion of the PEG chains, whereas RAS-LIF only relies on the depletion of the alkyl constituents. For the IL mixtures studied here, this would be quite challenging, as the PEG chains also contain CH_x_ groups in the chains that cannot be simply differentiated from the octyl chains. One other advantage of our study is that the investigation of surface composition and ST over a wide temperature range and under well-defined vacuum conditions allows for collecting very reliable data sets.

## 3. Conclusions

We investigated the macroscopic property surfaces tension and the microscopic surface composition of the newly synthesised and characterised IL [(mPEG_2_)_2_Im]I, of the well-studied IL [C_8_C_1_Im][PF_6_], and in particular of mixtures of these two ILs under ultra-clean vacuum conditions. Mixing common and well-characterized ILs is a relatively simple way to obtain new and fine-tuned IL systems for targeted applications. In particular, for large surface-area systems, which are used, e.g., in gas separation and adsorption technologies, film coatings, and catalysis, an understanding of surface composition and the related properties plays a crucial role.

The surface tension values of the pure ILs and their mixtures were measured using a new pendant drop setup, which allowed for measuring the ST in vacuum, and, thereby, minimised possible surface-active contaminations affecting the ST. The surface compositions were determined from ARXP spectra of all elements but hydrogen in UHV. ARXPS also allowed for verifying the ultraclean nature of the studied ILs. Both PD and ARXPS measurements were performed for the pure ILs and mixtures with about 5, 10, 20, and 50 mol% [C_8_C_1_Im][PF_6_] in the temperature range from about 300 to 360 K.

The ST of the pure ILs are found to be quite different, that is, 46.72 mN/m for pure [(mPEG_2_)_2_Im]I and 34.18 mN/m for [C_8_C_1_Im][PF_6_]. The values for the mixtures fall in between but strongly deviate from simple ideal mixing rules. From ARXPS, we deduce that the alkyl chain of pure [C_8_C_1_Im][PF_6_] and the PEG chains of pure [(mPEG_2_)_2_Im]I are enriched at the IL/vacuum interface. For the surface composition of the mixtures, a strong deviation from an ideal mixing behaviour also takes place with a strong selective enrichment of the alkyl chains for all mixtures, which is most pronounced at the lowest [C_8_C_1_Im][PF_6_] content. By applying a simple Langmuir approach based on the surface composition as deduced from ARXPS, we are able to predict and to reproduce the experimentally measured surface tension with astonishingly high accuracy. The comparison of the ST values obtained by PD measurements and those obtained from ARXPS measurements based on this simple Langmuir principle shows very good agreement in the case of the IL mixtures in our proof-of-concept model study. On the one hand, the information obtained from ARXPS thus provides a microscopic explanation for the macroscopic surface tension. On the other hand, one can now go a step further and deduce information on surface enrichment effects in mixtures from the deviation of the ST data obtained by the PD method from the ideal behaviour. While this certainly cannot fully replace using very complex and costly microscopic methods such as ARXPS, PD measurements nevertheless can provide important insights. This is particularly true for conditions such as high temperatures or gas pressures (even reactive conditions) where UHV-based methods cannot be applied.

## 4. Experimental Section

### 4.1. Materials

Angle-resolved X-ray photoelectron spectroscopy (ARXPS) and surface tension (ST) measurements were performed on 1-methyl-3-octyl-imidazolium hexafluorophosphate [C_8_C_1_Im][PF_6_] and 1,3-*bis*(polyethylene glycol)imidazolium iodide [(mPEG_2_)_2_Im]I, and mixtures thereof with 4.5, 9.6, 19.4, and 49.8 mol% [C_8_C_1_Im][PF_6_] content. [C_8_C_1_Im][PF_6_] (purity 99%) was purchased from IoLiTec (Heilbronn, Germany) and used as delivered, and [(mPEG_2_)_2_Im]I was synthesised as described in Ref. [50]. The structure of both ILs is shown in Figure 1. Mixtures were prepared in total amounts of one to three grams by weighing [C_8_C_1_Im][PF_6_] into [(mPEG_2_)_2_Im]I, swirling, and waiting overnight; the weights are provided in Table S4 in the Supplementary Materials). 

For the ARXPS measurements, each liquid sample was placed as a planar film of about 0.5 mm thickness on a molybdenum sample holder. Thereafter, sample degassing was performed in the fast entry load-lock of the vacuum chamber for at least 12 h. The ARXPS measurements were performed under ultra-high vacuum (UHV) at temperatures from 363 K down to around 240 K (and slightly below), where the onset of sample charging in ARXPS indicated solidification (see Table S4) [49]. The ST measurements were carried out under high vacuum (HV) conditions of ~10^−6^ mbar background pressure in a separate chamber [46], where the ILs and IL mixtures were first carefully degassed at a temperature of about 373 K for 15 h and then measured also from 363 K down to room temperature.

### 4.2. Angle-Resolved X-ray Photoelectron Spectroscopy

ARXPS was performed using our unique DASSA (dual analyser system for surface analysis) system; for details, see Ref. [49]. The measurements were performed with a monochromatic Al Kα source (*hν* = 1486.6 eV) at a power of 238 W. Spectra were acquired simultaneously with two ARGUS-type analysers positioned at ϑ = 0° (normal emission) and ϑ = 80° (grazing emission) with respect to the surface normal of the horizontally mounted sample. The information depth (ID) in 0° emission is 7 to 9 nm yielding predominantly information on the IL bulk. At 80° emission, the ID decreases by a factor of about six to 1 to 1.5 nm, yielding highly surface-sensitive information [49]. The temperature of the sample was measured directly at the IL-filled molybdenum reservoir with uncertainty in absolute temperature of ±2 K in the applied temperature window. High-resolution spectra were recorded with a pass energy of 35 eV to ensure an energy resolution of 0.4 eV. Survey scans were recorded with a pass energy of 150 eV. 

The XPS data were analysed using the CasaXPS software (version 2.3.16Dev6, Casa Software Ltd., Teignmouth, United Kingdom). For all peaks, a two-point linear background was subtracted, with the exception of the I 3d region, where a three-point linear background was set. Fitting was performed using a Gauss--Lorentzian function with a 30% Lorentzian contribution. For the quantitative analysis of the core levels, atomic sensitivity factors (ASFs) were applied [53]. For the spin-orbit split P 2p_1/2_ and 2p_3/2_ core levels, the intensity ratio was set to 1:2, with a peak separation of 0.9 eV and equal full width at half maximum (FWHM). For pure [C_8_C_1_Im][PF_6_], the C 1s region was fitted with three peaks for C_2_, C_hetero,_ and C_alkyl_. The C_2_ and C_hetero_ peaks are separated by 0.9 eV, with an intensity ratio of 1:4 and FWHMs of 1:1.1; C_2_ and C_alkyl_ have the same FWHM. For [(mPEG_2_)_2_Im]I, the C 1s region was fitted with two peaks, C_2_ and C_hetero_, with peak separation of 0.87 eV and a ratio of 1:12. The FWHM of C_hetero_ was 1.19 times that of the C_2_ peak. The C 1s region of the mixtures was fitted by considering the different peak positions, peak widths of the pure ILs, and the corresponding mixing ratios and resulting atomic ratios; since the C_alkyl_ signals in the mixtures are small for low [C_8_C_1_Im][PF_6_] content, we used an additional constraint for unambiguous fitting, namely a fixed peak separation of the C_hetero_ and the C_alkyl_ peaks of 1.59 eV in 0° and 1.46 eV in 80° (these values were derived at 298 K from the 1:1 mixture, where the two peaks were clearly discernible, and varied by ±0.05 eV with temperature—see Table S5 in the Supplementary Materials; an exemplary fit of the 298 K C 1s spectrum measured in 0° is shown in Figure S1 in the Supplementary Materials; the resulting binding energies and the quantitative analysis of all ARXPS data at 298 K are provided in Table S6 in the Supplementary Materials).

To allow for visualising depletion/enrichment effects through direct comparison, all 80° measurements were scaled up by a geometry factor, which is derived from the sum of the weighted intensities of all core levels obtained in 0° and 80°, following a previously established procedure [15,49]. Moreover, the energy scales of all 80° spectra were slightly shifted by −0.1 eV such that the N 1s peaks in 80° of the common imidazolium head group fall onto the N 1s peaks in 0°.

### 4.3. High Vacuum Pendant Drop Measurements

Surface tension measurements were performed in a high vacuum pendant drop (PD) system, which was recently developed by our group. The properties and calibration of the setup were described in a recent publication [46]. The applied stainless steel cannula has an outer diameter of 2.02 mm. Videos of the drops were recorded with a high-speed camera (4 frames per second). For the evaluation of the ST, 10 frames were used per droplet. To ensure the reproducibility of the data, 5 droplets were formed at a fixed temperature (with a maximum temperature drift of ±1 K). The ST was determined from each droplet shape using the axisymmetric drop shape analysis—(ADSA) and the Young--Laplace equation solution—software package SCA 22/15 (version 5.0.35) from DataPhysics (V.5.0.35build5035, DataPhysics Instruments GmbH, Filderstadt, Germany); for further details on the setup and evaluation procedure, see Ref. [46].

## Figures and Tables

**Table 1:** Coefficients of the linear fits to the data according to Equation (1) for pure ILs and mixtures and ST values at 298 K.

	[C_8_C_1_Im][PF_6_]	49.8 mol%	19.4 mol%	9.6 mol%	4.5 mol%	[(mPEG_2_)_2_Im]I
$\gamma_{0}$ [mN/m]	50.67	53.54	57.89	61.44	64.84	72.22
$\gamma_{1}$ [mN/m·K^−1^]	−0.0553	−0.0530	−0.0560	−0.0613	−0.0677	−0.0855
$\gamma_{298\;K}$ [mN/m]	34.18	37.72	41.20	43.16	44.67	46.72

**Table 2:** Experimental ST values γ for different mixtures and temperatures (as derived from linear fits to the data according to Equation (1) with coefficients from Table 1), along with calculated ST values γ_80°_(calc) using Equation (3) and the ARXPS 80° results shown in Figure 6b (see also bottom row of Table S2).

	49.8 mol%		19.4 mol%		9.6 mol%		4.5 mol%	
T [K]	γ [mN/m]	γ_80°_(calc) [mN/m]	γ [mN/m]	γ_80°_(calc) [mN/m]	γ [mN/m]	γ_80°_(calc) [mN/m]	γ [mN/m]	γ_80°_(calc) [mN/m]
363	34.28	34.24	37.56	37.35	39.17	38.72	40.27	40.29
348	35.07	35.06	38.40	38.19	40.09	39.80	41.29	41.30
323	36.40	36.32	39.80	39.45	41.63	42.34	42.98	43.00
298	37.72	37.75	41.20	40.36	43.16	42.45	44.67	44.32

## Data Availability

The data presented in this study are available on request from the corresponding author.

## Supplementary Materials

The following supplementary material can be downloaded at: https://www.mdpi.com/article/10.3390/molecules27238561/s1, Figure S1:Exemplary fitting procedure for the C 1s region of the 49.8 mol% [C_8_C_1_Im][PF_6_] content mixture in 0° at T = 298 K; Figure S2: F 1s, I 3d_5/2_, O 1s, N 1s, C 1s and P 2p spectra at 0° and 80° emission of pure [C_8_C_1_Im][PF_6_], pure [(mPEG_2_)_2_Im]I, and of different mixtures of these ILs, collected at T = 298 K; Figure S3: Composition ratios n(80°)/n_nominal_ for F 1s, O_PEG_, I 3d_5/2_, N 1s, C_2_, C_hetero_, C_alkyl_ and P 2p, derived from the corresponding 80° intensities and their nominal atomic numbers for the neat ILs and mixtures for different temperatures; Figure S4: Comparison of the linear fits of the experimental data determined using the pendant drop method in vacuum with the surface tension as expected for ideal behaviour of different mixtures; Figure S5: (a) Surface tension values of the pure ILs and the mixtures at 298 K, as determined from pendant drop measurements in Figure 5, along with the surface tension values calculated from the 0° ARXPS data according to Equation (3). For comparison, the surface tension of ideal mixtures without enrichment/depletion effects is shown as black line. (b) Number of atoms, n(0°), as determined from the ARXPS data at 0°, normalized to the number of corresponding atoms in the IL for O_PEG_ and for C_alkyl_ for the pure ILs and for the mixtures at T = 298 K; Figure S6: Comparison of the experimentally determined surface tension values derived from the pendant drop measurements in vacuum with the surface tension expected for ideal behaviour of the different mixtures. Also shown are the predictions of the surface tension from the ARXPS data at 0°; Table S1: Temperature-dependent density parameters for pure [C_8_C_1_Im][PF_6_] from Ref. [54], [(mPEG_2_)_2_Im]I from Ref. [50] and their mixtures with 4.5, 9.6, 19.4 and 49.8 mol% [C_8_C_1_Im][PF_6_] content; the densities for the surface tension evaluation at different temperatures are obtained following ρ = ρ_0_ + ρ_1_·T + ρ_2_·T^2^ (temperature in K);Table S2: Number of O_PEG_ and C_alkyl_ atoms derived from the temperature-dependent 0° and 80° ARXPS spectra; Table S3: ST values γ(ideal) for different mixtures and temperatures as expected from ideal mixing behaviour using Equation (2) along calculated ST values γ_0°_(calc.) using Equation (3) with the number of O_PEG_ and C_alkyl_ atoms as deduced from the 0° ARXP spectra; Table S4: Weights of [C_8_C_1_Im][PF_6_] and [(mPEG_2_)_2_Im]I used for the 4.5, 9.6, 19.4 and 49.8 mol% mixtures; last column shows solidification temperatures of the corresponding mixtures as indicated by the onset of sample charging in ARXPS; Table S5: Binding energy (BE) differences of the C_hetero_ and C_alkyl_ peaks for both angles as a function of temperature of the 49.8 mol% mixture. These ΔBE values were used as constraints to fit the C 1s spectra of all other mixtures; Table S6: Quantitative analysis of the 0° and 80° ARXPS spectra for pure [C_8_C_1_Im][PF_6_], [(mPEG_2_)_2_Im]I and their mixtures with 4.5, 9.6, 19.4 and 49.8 mol% [C_8_C_1_Im][PF_6_] content at 298 K. Reference [54] are cited in the supplementary materials.

## Sample Availability

Samples of the compounds are not available from the authors.
