# Supplementary material for: Direct Correlation of Surface Tension and Surface Composition of Ionic Liquid Mixtures—A Combined Vacuum Pendant Drop and Angle-Resolved X-ray Photoelectron Spectroscopy Study
Source: Molecules. 2022 Dec 5;27(23):8561. doi: 10.3390/molecules27238561 (PMC9736233; doi:10.3390/molecules27238561)
Supplement: Supplementary file 1 [file molecules-27-08561-s001.zip › molecules-2055421-supplementary.pdf]

Supplementary Materials

Direct Correlation of Surface Tension and Surface Composition of Ionic Liquid Mixtures – A Combined Vacuum Pendant Drop and Angle-Resolved X-Ray Photoelectron Spectroscopy Study

Ulrike Paap *ORCID: 0000-0002-1442-0239 [a]*, Vera Seidl *ORCID: 0000-0003-3310-700X [b]*,
Karsten Meyer *ORCID: 0000-0002-7844-2998 [b]*, Florian Maier *ORCID: 0000-0001-9725-8961 [a]*,
and Hans-Peter Steinrück* *ORCID: 0000-0003-1347-8962 [a]*

[a] Department of Chemistry and Pharmacy, Physical Chemistry II, Friedrich-Alexander-Universität Erlangen-Nürnberg (FAU), Egerlandstr. 3, 91058 Erlangen

[b] Department of Chemistry and Pharmacy, Inorganic Chemistry, Friedrich-Alexander-Universität Erlangen-Nürnberg (FAU), Egerlandstr. 1, 91058 Erlangen

(* hans-peter.steinrueck@fau.de)

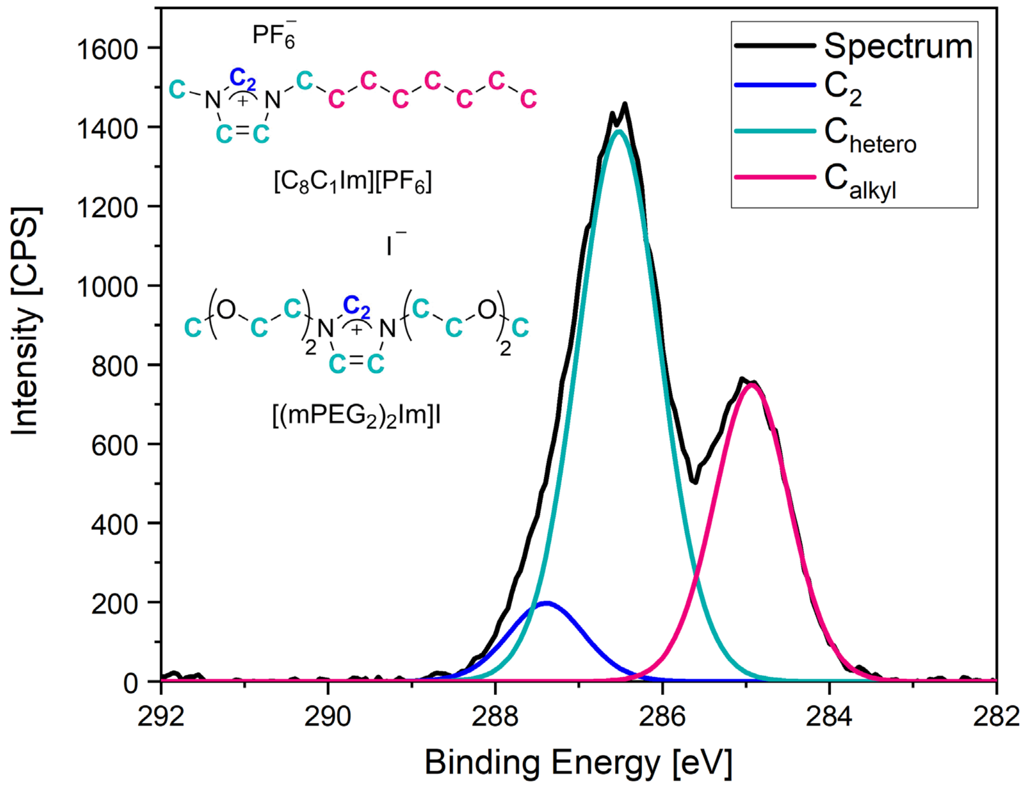

Figure S1: *Exemplary fitting procedure for the C 1s region of the 49.8 mol% [C_8C_1Im][PF_6] content mixture in 0° at T = 298 K. The spectrum (black) is fitted with peaks for C_2 (blue), C_{hetero} (turquoise) and C_{alkyl} (pink) carbon atoms (see inset).*

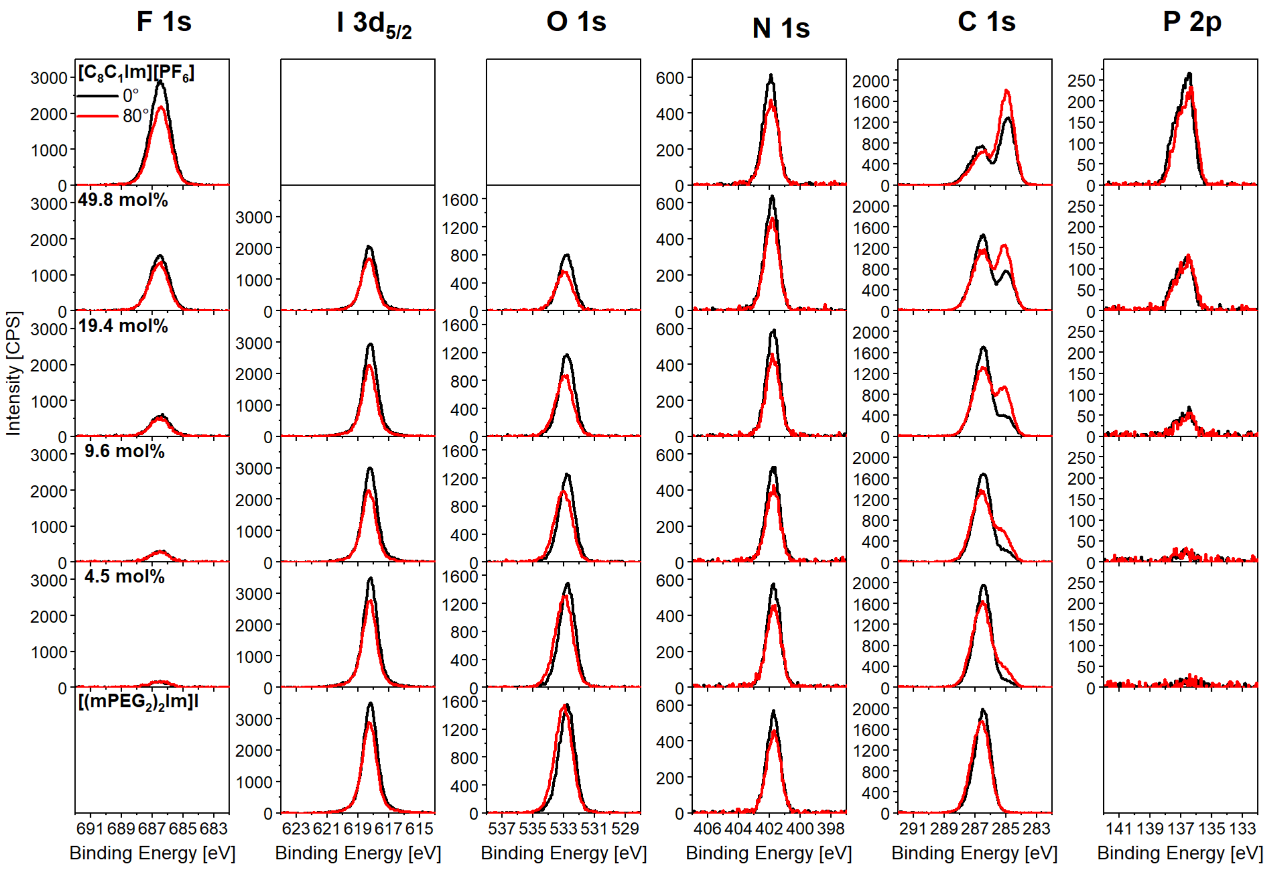

Figure S2: *F 1s, I 3d$_{5/2}$, O 1s, N 1s, C 1s and P 2p spectra at 0° (black) and 80° (red) emission of pure [C_8C_1Im][PF_6] (top row), pure [(mPEG$_2$)$_2$Im]I (bottom row), and of different mixtures of these ILs, collected at T = 298 K.*

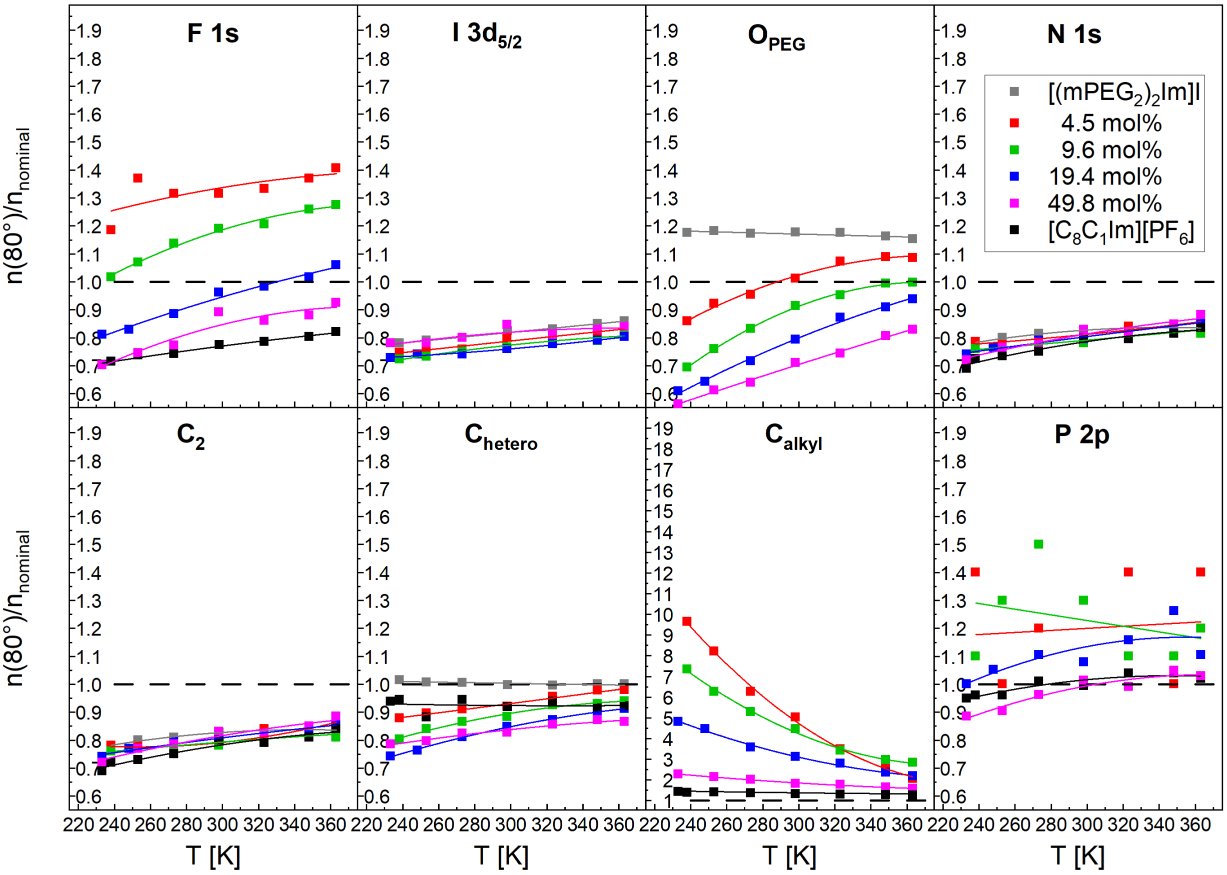

Figure S3: *Composition ratios n(80°)/$n_{nominal}$ for F 1s, O_{PEG}, I $3d_{5/2}$, N 1s, C_2, C_{hetero}, C_{alkyl} and P 2p, derived from the corresponding 80° intensities and their nominal atomic numbers for the neat ILs and mixtures for different temperatures (note the different scaling for the C_{alkyl} signal).*

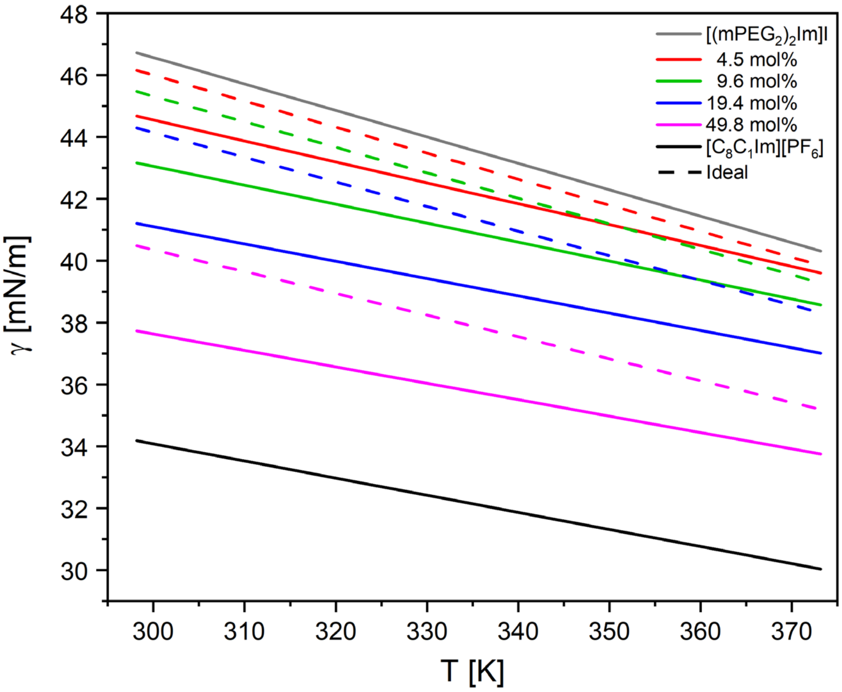

Figure S4: *Comparison of the linear fits (solid lines) of the experimental data determined using the pendant drop method in vacuum (Figure 5 and equation (1) in the manuscript) with the surface tension as expected for ideal behaviour of different mixtures (dashed lines). Clearly, the measured surface tension of all mixtures and temperatures strongly deviates from the ideal mixing rules.*

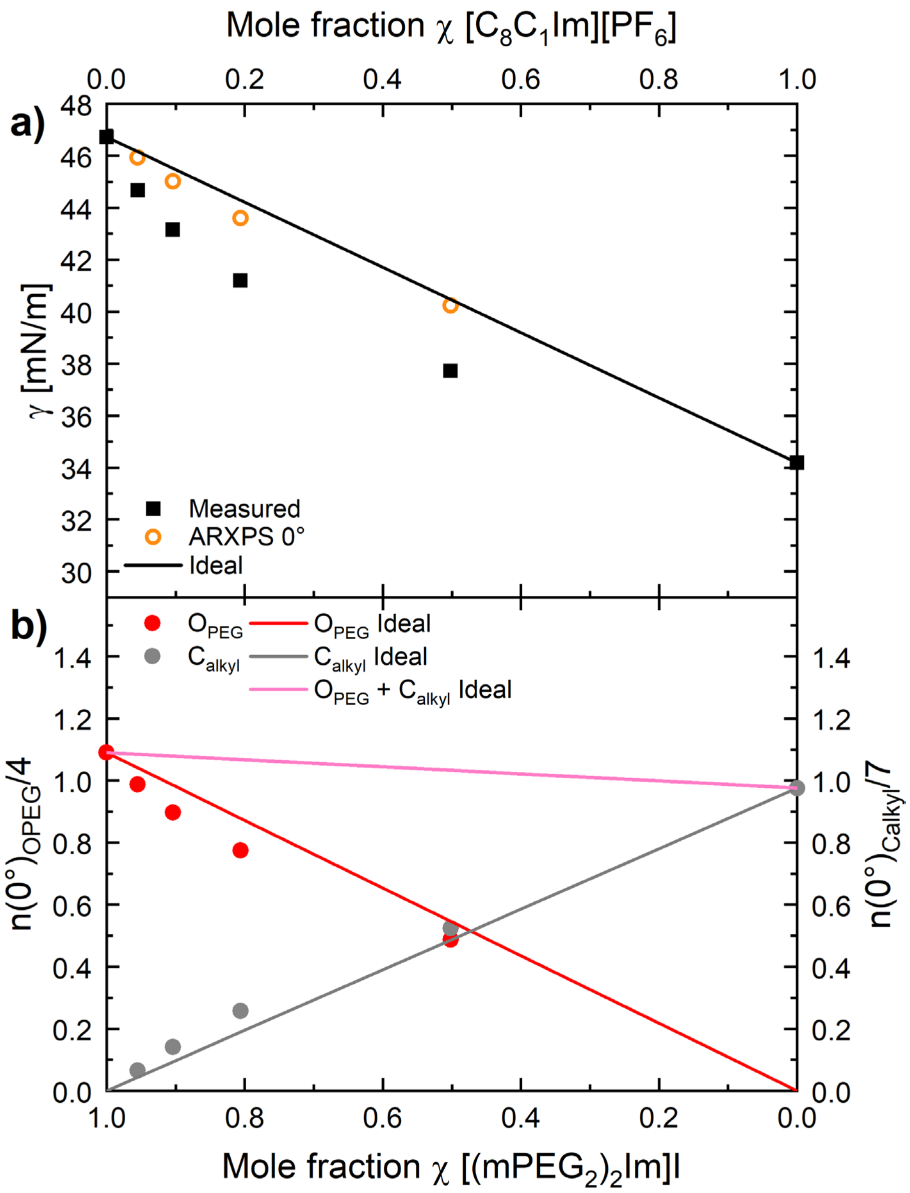

Figure S5: *a) Surface tension values of the pure ILs and the mixtures at 298 K (black squares), as determined from pendant drop measurements in Figure 5, along with the surface tension values calculated from the 0° ARXPS data according to equation (3) (open orange circles). For comparison, the surface tension of ideal mixtures without enrichment/depletion effects is shown as black line. b) Number of atoms, n(0°), as determined from the ARXPS data at 0°, normalized to the number of corresponding atoms in the IL for* O_{PEG} *(red circles) and for* C_{alkyl} *(grey circles) for the pure ILs and for the mixtures at T = 298 K. Lines represent the ideal bulk mixing behaviour without enrichment/depletion effects at the surface. In contrast to the 80° ARXPS data that reproduce the measured surface tension values (see Figure 6a), the 0° data follow the ideal behaviour as expected due to their reduced surface sensitivity.*

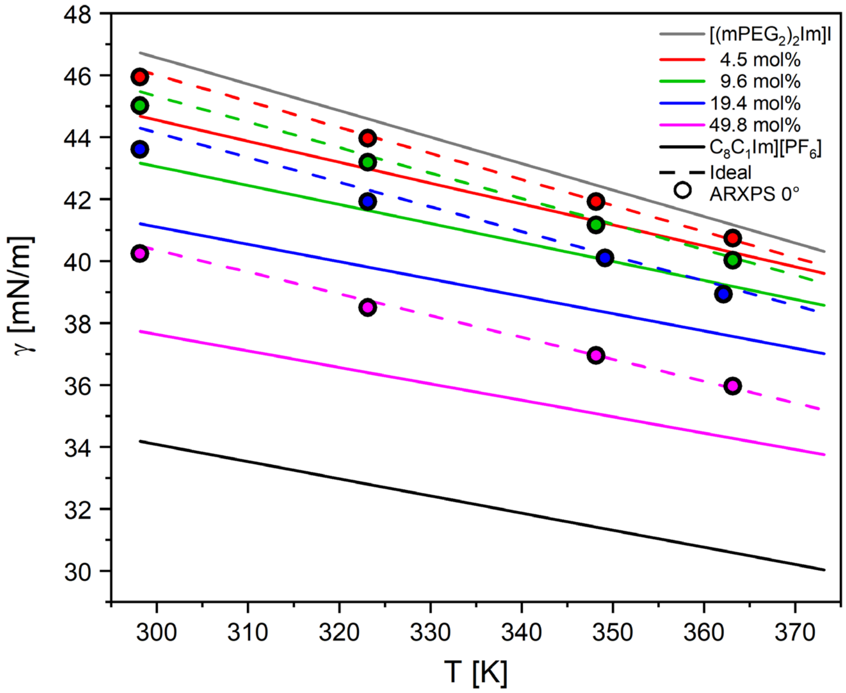

Figure S6: *Comparison of the experimentally determined surface tension values derived from the pendant drop measurements in vacuum (solid lines as linear fits to the experimental data, see Figure 5 and equation (1) in the manuscript) with the surface tension expected for ideal behaviour of the different mixtures (dashed lines). Also shown are the predictions of the surface tension from the ARXPS data at 0° (filled circles, for details, see text). For each mixture, the surface tension values predicted from ARXPS at 0° strongly deviate from the pendant drop results (solid lines), but follow the expected ideal behaviour (dashed lines, see equation (2)).*

Table S1: *Temperature-dependent density parameters for pure [C_8C_1Im][PF_6] from Ref. [1], [(mPEG$_2$)$_2$Im]I from Ref. [2] and their mixtures with 4.5, 9.6, 19.4 and 49.8 mol% [C_8C_1Im][PF_6] content; the densities for the surface tension evaluation at different temperatures are obtained following* $\rho = \rho_0 + \rho_1 \cdot T + \rho_2 \cdot T^2$ *(temperature in K).*

	ρ_0 [g/cm^3]	ρ_1 [g/cm^3·K]	ρ_2 [g/cm^3·K^2]
**[C_8C_1Im][PF_6]**	1.61	$-1.59\cdot10^{-3}$	$1.21\cdot10^{-6}$
**49.8 mol%**	1.65	$-1.30\cdot10^{-3}$	$7.39\cdot10^{-7}$
**19.4 mol%**	1.69	$-1.29\cdot10^{-3}$	$6.40\cdot10^{-7}$
**9.6 mol%**	1.71	$-1.22\cdot10^{-3}$	$6.07\cdot10^{-7}$
**4.5 mol%**	1.72	$-1.21\cdot10^{-3}$	$5.91\cdot10^{-7}$
**[(mPEG$_2$)$_2$Im]I**	1.72	$-1.20\cdot10^{-3}$	$5.70\cdot10^{-7}$

Table S2: *Number of O_{PEG} and C_{alkyl} atoms derived from the temperature-dependent 0° and 80° ARXPS spectra.*

**0°**								
	**49.8 mol%**		**19.4 mol%**		**9.6 mol%**		**4.5 mol%**	
T [K]	$n_{OPEG,0°}(x)$	$n_{Calkyl,0°}(x)$	$n_{OPEG,0°}(x)$	$n_{Calkyl,0°}(x)$	$n_{OPEG,0°}(x)$	$n_{Calkyl,0°}(x)$	$n_{OPEG,0°}(x)$	$n_{Calkyl,0°}(x)$
363	2.04	3.46	3.22	1.51	3.71	0.79	4.09	0.31
348	2.01	3.50	3.20	1.52	3.69	0.85	40.5	0.36
323	1.95	3.65	3.18	1.63	3.65	0.86	4.04	0.40
298	1.95	3.67	3.10	1.80	3.59	0.99	3.95	0.46
**80°**								
	**49.8 mol%**		**19.4 mol%**		**9.6 mol%**		**4.5 mol%**	
T [K]	$n_{OPEG,80°}(x)$	$n_{Calkyl,80°}(x)$	$n_{OPEG,80°}(x)$	$n_{Calkyl,80°}(x)$	$n_{OPEG,80°}(x)$	$n_{Calkyl,80°}(x)$	$n_{OPEG,80°}(x)$	$n_{Calkyl,80°}(x)$
363	1.67	5.53	3.02	2.98	3.61	1.90	4.15	0.66
348	1.62	5.75	2.93	3.22	3.60	1.99	4.16	0.85
323	1.50	6.18	2.81	3.80	3.45	2.30	4.10	1.12
298	1.43	6.34	2.55	4.23	3.31	3.00	3.87	1.61

Table S3: *ST values γ (ideal) for different mixtures and temperatures as expected from ideal mixing behaviour using equation (2) along calculated ST values $\gamma_{0°}$(calc.) using equation (3) with the number of O_{PEG} and C_{alkyl} atoms as deduced from the 0° ARXP spectra (top row of Table S5).*

	**49.8 mol%**		**19.4 mol%**		**9.6 mol%**		**4.5 mol%**	
**T [K]**	*γ (ideal) [mN/m]*	*$\gamma_{0°}$(calc.) [mN/m]*	*γ (ideal) [mN/m]*	*$\gamma_{0°}$(calc.) [mN/m]*	*γ (ideal) [mN/m]*	*$\gamma_{0°}$(calc.) [mN/m]*	*γ (ideal) [mN/m]*	*$\gamma_{0°}$(calc.) [mN/m]*
**363**	35.90	35.96	39.11	38.93	40.11	40.02	40.68	40.73
**348**	36.96	36.95	40.30	40.10	41.34	41.17	41.95	41.92
**323**	38.72	38.50	42.30	41.92	43.41	43.19	44.05	43.96
**298**	40.48	40.24	44.29	43.60	45.47	45.02	46.15	45.94

Table S4: *Weights of $[C_8C_1Im][PF_6]$ and $[(mPEG_2)_2Im]I$ used for the 4.5, 9.6, 19.4 and 49.8 mol% mixtures; last column shows solidification temperatures of the corresponding mixtures as indicated by the onset of sample charging in ARXPS.*

	**$[C_8C_1Im][PF_6]$ [g]**	**$[(mPEG_2)_2Im]I$ [g]**	**Solidification Temperature [K]**
**4.5 mol%**	0.0424	1.0486	237
**9.6 mol%**	0.2476	2.7352	235
**19.4 mol%**	0.1624	0.7992	231
**49.8 mol%**	1.0257	1.2193	230

Table S5: *Binding energy (BE) differences of the C_{hetero} and C_{alkyl} peaks for both angles as a function of temperature of the 49.8 mol% mixture. These ΔBE values were used as constraints to fit the C 1s spectra of all other mixtures (for details, see text).*

T [K]	ΔBE(0°) [eV]	ΔBE(80°) [eV]
**363**	1.62	1.49
**348**	1.61	1.48
**323**	1.61	1.47
**298**	1.59	1.46
**273**	1.57	1.42
**253/248**	1.54	1.40
**238/233**	1.53	1.39

Table S6: *Quantitative analysis of the 0° and 80° ARXPS spectra for pure [C_8C_1Im][PF_6], [(mPEG$_2$)$_2$Im]I and their mixtures with 4.5, 9.6, 19.4 and 49.8 mol% [C_8C_1Im][PF_6] content at 298 K.*

	**F 1s**	**I 3d$_{5/2}$**	**O 1s**	**N 1s**	**C_2**	**C_{hetero}**	**C_{alkyl}**	**P 2p**
ASF	1.00	6.17	0.67	0.46	0.3	0.3	0.3	0.46
**[C_8C_1Im][PF_6]**								
0° BE/eV	686.5			401.9	287.5	286.6	284.9	137.4
80° BE/eV	686.7			402.1	287.6	286.7	285.1	137.4
Nominal	***6***			***2***	***1***	***4***	***7***	***1***
n(0°)	6.11			1.99	1.00	3.97	6.84	1.09
n(80°)	4.62			1.60	0.80	3.61	9.38	0.99
**49.8 mol%**								
0° BE/eV	868.6	618.3	532.9	401.9	287.4	286.5	284.9	137.5
80° BE/eV	868.7	618.4	533.1	402.0	287.5	286.6	285.2	137.5
Nominal	***2.99***	***0.50***	***2.01***	***2.00***	***1.00***	***8.02***	***3.48***	***0.50***
n(0°)	3.14	0.53	1.96	1.99	0.99	7.61	3.75	0.52
n(80°)	2.70	0.44	1.48	1.69	0.85	6.68	6.15	0.52
**19.4 mol%**								
0° BE/eV	686.5	618.2	532.8	401.8	287.3	286.5	284.9	137.4
80° BE/eV	686.6	618.4	533.1	401.9	287.5	286.6	285.1	137.4
Nominal	***1.17***	***0.81***	***3.22***	***2.00***	***1.00***	***10.5***	***1.36***	***0.19***
n(0°)	1.30	0.82	3.12	1.98	0.99	9.89	1.85	0.25
n(80°)	1.12	0.62	2.56	1.56	0.78	8.80	4.54	0.21
**9.6 mol%**								
0° BE/eV	686.5	618.2	532.8	401.8	287.3	286.5	284.9	137.5
80° BE/eV	686.7	618.4	533.2	401.9	287.6	286.7	285.2	137.6
Nominal	***0.58***	***0.90***	***3.62***	***2.00***	***1.00***	***11.2***	***0.67***	***0.10***
n(0°)	0.70	0.90	3.59	1.95	0.98	10.9	0.99	0.11
n(80°)	0.69	0.69	3.31	1.56	0.78	9.93	3.00	0.13
**4.5 mol%**								
0° BE/eV	868.6	618.2	532.8	401.7	287.3	286.5	284.9	137.1
80° BE/eV	686.7	618.4	533.1	401.8	287.5	286.6	285.2	137.3
Nominal	***0.27***	***0.95***	***3.82***	***2.00***	***1.00***	***11.6***	***0.32***	***0.05***
n(0°)	0.33	0.96	3.96	1.91	0.96	11.4	0.48	0.08
n(80°)	0.40	0.76	3.94	1.64	0.82	10.8	1.63	
**[(mPEG$_2$)$_2$Im]I**								
0° BE/eV		618.2	532.8	401.7	287.3	286.5		
80° BE/eV		618.4	533.2	401.9	287.6	286.7		
Nominal		***1***	***4***	***2***	***1***	***12***		
n(0°)		1.00	4.34	1.94	0.97	11.7		
n(80°)		0.84	4.74	1.66	0.83	11.9		

References:

1. Gardas, R.L.; Freire, M.G.; Carvalho, P.J.; Marrucho, I.M.; Fonseca, I.M.A.; Ferreira, A.G.M.; Coutinho, J.A.P. High-Pressure Densities and Derived Thermodynamic Properties of Imidazolium-Based Ionic Liquids. *Journal of Chemical & Engineering Data* **2007**, *52*, 80-88, doi:10.1021/je060247x.
2. Seidl, V.; Bosch, M.; Paap, U.; Livraghi, M.; Zhai, Z.; Wick, C.R.; Koller, T.M.; Wasserscheid, P.; Maier, F.; Smith, A.-S.; et al. Bis-polyethylene glycol-functionalized imidazolium ionic liquids: A multi-method approach towards bulk and surface properties. *Journal of Ionic Liquids* **2022**, *2*, 100041, doi:10.1016/j.jil.2022.100041.
